# Understanding Mucormycoses in the Age of “omics”

**DOI:** 10.3389/fgene.2020.00699

**Published:** 2020-06-30

**Authors:** Alexandra Y. Soare, Tonya N. Watkins, Vincent M. Bruno

**Affiliations:** ^1^Department of Microbiology and Immunology, University of Maryland School of Medicine, Baltimore, MD, United States; ^2^Institute of Genome Sciences, University of Maryland School of Medicine, Baltimore, MD, United States

**Keywords:** genomics, transcriptomics, mucormycosis, RNA-seq, WGS, emerging fungal disease

## Abstract

Mucormycoses are deadly invasive infections caused by several fungal species belonging to the subphylum Mucoromycotina, order *Mucorales*. Hallmarks of disease progression include angioinvasion and tissue necrosis that aid in fungal dissemination through the blood stream, causing deeper infections and resulting in poor penetration of antifungal agents to the site of infection. In the absence of surgical removal of the infected focus, antifungal therapy alone is rarely curative. Even when surgical debridement is combined with high-dose antifungal therapy, the mortality associated with mucormycoses is >50%. The unacceptably high mortality rate, limited options for therapy and the extreme morbidity of highly disfiguring surgical therapy provide a clear mandate to understand the molecular mechanisms that govern pathogenesis with the hopes of developing alternative strategies to treat and prevent mucormycoses. In the absence of robust forward and reverse genetic systems available for this taxonomic group of fungi, unbiased next generation sequence (NGS)-based approaches have provided much needed insights into our understanding of many aspects of Mucormycoses, including genome structure, drug resistance, diagnostic development, and fungus-host interactions. Here, we will discuss the specific contributions that NGS-based approaches have made to the field and discuss open questions that can be addressed using similar approaches.

## Introduction

Mucormycoses are increasingly common, life-threatening, invasive fungal infections (IFI) that are caused by various fungal species belonging to the subphylum Mucoromycotina, order *Mucorales* (Ribes et al., 2000; Spellberg et al., 2005). *Mucorales* are fast-growing, thermotolerant fungi that are ubiquitous in soil, on fruit, dust, and decaying vegetation worldwide (Rogers, 2008; Petrikkos et al., 2012). They are commonly found in homes and one study indicated that *Mucorales* species were present in 98% of samples taken from home dust (Gravesen, 1978; Quandahl and Cooper, 2018). *Mucorales* are considered opportunistic pathogens, requiring a suppressed immune system or another underlying condition to cause disease, and are the third most common cause of IFIs in immunocompromised patients (Michael et al., 2006; Rogers, 2008; Tacke et al., 2014; Millon et al., 2015). Fatal mucormycosis infections can be initiated by inhalation, ingestion, or contamination of wounds with easily aerosolized spores from the environment (Michael et al., 2006).

Mucormycoses are associated with high morbidity and mortality, >50% and approaching 100% with disseminated infection despite aggressive tissue debridement and antifungal therapy (Puebla, 2012; Katragkou et al., 2014). Generally, mucormycoses will spread widely and cause extensive tissue damage by the time infection is diagnosed (Puebla, 2012; Katragkou et al., 2014). *Mucorales* establish infection in immunocompromised individuals with predisposing risk factors including uncontrolled diabetes resulting in hyperglycemia and ketoacidosis (DKA), chemotherapy, hematological disease, organ transplantation, elevated blood iron, deferoxamine or corticosteroid therapy, among others (Ghuman and Voelz, 2017). *Mucorales* can also cause lethal infections in a broader and more heterogeneous population than other opportunistic molds including injection drug users, patients receiving prolonged antifungal treatment lacking activity against *Mucorales* (i.e., Voriconazole), and those exposed to recent hospital construction (Michael et al., 2006; Rammaert et al., 2012; Lewis et al., 2012; Bernal-Martinez et al., 2013). Immunocompetent victims of natural disasters (earthquakes, tsunamis, tornados, etc.) and traumatic accidents such as those resulting from burns and military-related combat are also susceptible to mucormycosis (Ibrahim et al., 2012; Ibrahim and Kontoyiannis, 2013).

There are currently 27 different *Mucorales* species, across 11 genera, that have been identified as a causative agent of mucormycosis (Roden et al., 2005; Gomes et al., 2011; Jeong et al., 2019; Walther et al., 2019b). Whole genome sequences are available for 21 of the 27^1^. *Rhizopus* species are the most common cause, accounting for ∼70% of all cases and are the most common organisms isolated from patients with mucormycosis (Ribes et al., 2000; Roden et al., 2005; Spellberg et al., 2005; Ibrahim and Kontoyiannis, 2013; Gebremariam et al., 2014; Walther et al., 2019a). *Mucor* spp. and *Lichtheimia* spp. are also a significant cause of these fungal infections in Europe with each causing ∼20% of the cases (Skiada et al., 2011), while *Apophysomyces* spp. are common clinical isolates in India (Chakrabarti and Singh, 2014). In all, the number of mucormycosis incidences is increasing and is estimated to be 500 cases per year in the United States (Spellberg et al., 2005; Michael et al., 2006). A prospective surveillance study of nearly 17,000 transplant recipients performed in 23 institutions during 2001–2006 reported that mucormycosis was the third most common IFI in stem cell transplant recipients, with invasive aspergillosis (IA) and invasive candidiasis being the first and second most common, respectively (Kontoyiannis et al., 2010; Mucormycosis Statistics, 2018). The National Institute of Allergy and Infectious Disease (NIAID) now classifies mucormycosis as an emerging infectious disease (Chibucos et al., 2016; NIAID, 2018). Importantly, the true prevalence of mucormycoses is difficult to determine. Since there are no reporting requirements for fungal infections, no national surveillance in the United States, a lack of accurate diagnostic assays, and a declining rate of autopsies in high-risk populations, the true number of mucormycosis infections per year is likely to be severely underestimated (Lewis et al., 2012; Ibrahim and Kontoyiannis, 2013; Walsh et al., 2014; Mucormycosis Statistics, 2018).

**FIGURE 1:**
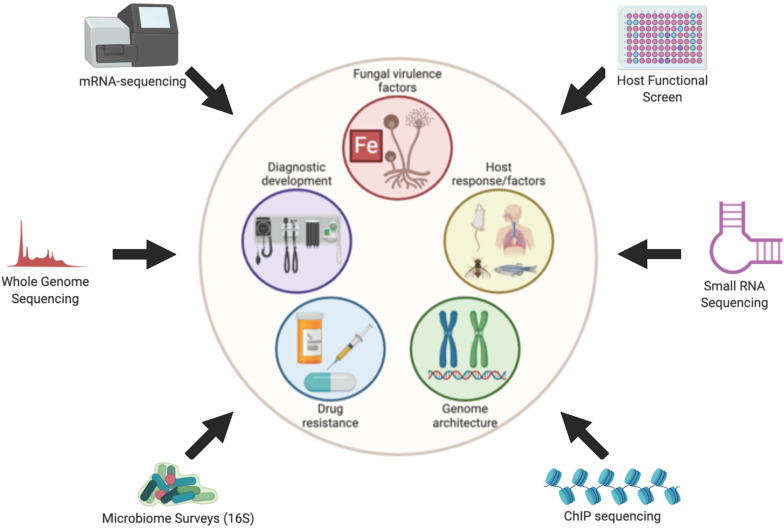
Overview of Omics approaches used to study Mucormycoses. Figure created with BioRender.com.

Very little is known about the molecular mechanisms that govern pathogenesis of *Mucorales*, compared to better studied fungal pathogens such as *Candida albicans*, *Cryptococcus neoformans*, and *Aspergillus fumigatus.* This knowledge gap is due, in large part, to the genetic intractability of the *Mucorales*. Furthermore, the ability to make educated guesses and form hypotheses about *Mucorales* pathogenesis based on molecular mechanisms proven in other fungal pathogens is limited by the large evolutionary distance that separates *Mucorales* from Ascomycetes (e.g., *Candida, Aspergillus*) and Basidiomycetes (e.g., *Cryptococcus*). Specifically, Mucormycetes, which include all *Mucorales*, are thought to have diverged from a common ancestor with Ascomycetes and Basidiomycetes over 800 million years ago (Galagan et al., 2005). Gene deletion strategies have been developed for *Mucor circinelloides* and RNAi-based knock-down approaches have been used to study *M. circinelloides* and *Rhizopus delemar*. These strategies have provided valuable insights into the molecular pathogenesis of mucormycoses, but their overall use has been limited to only a handful of studies (Ibrahim et al., 2010; Gebremariam et al., 2014; Liu et al., 2015; Trieu et al., 2017; Vellanki et al., 2018; Garcia et al., 2018). In the absence of robust forward and reverse genetic systems, unbiased next generation sequence (NGS)-based approaches have provided important insights and exploratory avenues into understanding, diagnosing and developing desperately needed therapies for this emerging class of infections. Here, we review the specific contributions that NGS-based approaches have made toward the overall knowledge and understanding of *Mucorales* pathogenesis (Figure 1) helping to lead to the development of therapies to treat this disease.

## Insights Into *Mucorales* Biology and Virulence Determinants

### Genome Architecture and Structure

Sequencing of the *R. delemar* stain 99–880 genome revealed a highly repetitive genome indicative of an ancestral whole-genome duplication (WGD) event, which resulted in the replication of gene families related to cell growth, signal transduction, and cell-wall synthesis (Ma et al., 2009). Similar patterns were seen in the genomes of *M. circinelloides* and *Phycomyces blakesleeanus*, a non-pathogenic member of the *Mucorales* order known for its phototrophic growth (Corrochano et al., 2016). Both fungi showed evidence of widespread genome duplication that was concurrent with *R. delemar*, suggesting that the WGD event occurred early in the Mucoromycotina subdivision lineage. Fungi in the mucoromycotina subdivision appear to have more duplicated regions than other fungal genomes; however, evidence of a WGD event is not found in all of them. While the whole genome sequence of *Lichtheimia corymbifera* indicates a high occurrence of gene duplication and expansion, there appears to be no evidence of WGD. Rather, the duplicative nature of the *L. corymbifera* genome appears to be mediated by the high occurrence of tandem duplications (Schwartze et al., 2014). This pattern was also observed in whole genome sequencing and comparison of *Apophysomyces* species, which demonstrated extensive gene duplication and expansion across its genome but no evidence of a WGD (Prakash et al., 2017).

Other relevant features of *Mucorales* genomes have been elucidated through genome sequencing. *Rhizopus* species demonstrate remarkable variety in genome length, notably within *Rhizopus microsporus* (Gryganskyi et al., 2018). Additionally, there is wide variety in the structure of the mating type locus within *Rhizopus* genomes that vary from typical arrangements seen in Mucoralean fungi (Gryganskyi et al., 2018). Similar to *L. corymbifera* (Schwartze et al., 2014), *Apophysomyces* species had a lower number of transposable elements (TEs) in their genome when compared to other *Mucorales* species (Prakash et al., 2017). Both cases were associated with multiple copies of heterokaryon incompatibility (HET) genes and genes associated with RNA interference (RNAi) pathway (Schwartze et al., 2014; Prakash et al., 2017).

Chromatin immunoprecipitation sequencing (ChIP-seq) of *M. circinelloides* showed that it has a unique “mosaic” centromere structure in Mucoromycotina, with characteristics from point centromeres (seen in *Saccharomyces cerevisiae*) and regional centromeres (seen in *C. albicans, Candida tropicalis, Magnaporthe oryzae, Schizosaccharomyces pombe, and C. neoformans*) (Navarro-Mendoza et al., 2019).

### *Mucorales* Growth and Metabolism

Various RNA-seq studies of *Mucorales* species have uncovered some basic principles regarding growth and metabolism. Little is known about the mechanisms behind spore germination, an important mechanism during filamentous growth as dormant spores transform to a vegetative state marked by hyphal growth. Germination and subsequent hyphal growth is responsible for the invasive nature of mucormycoses and can be initiated under multiple conditions. Sephton-Clark et al. (2018) performed a transcriptome analysis of *R. delemar* cells on a 24 h time course from dormant spores through germinating spores to full hyphal growth. Naturally, RNA from *R. delemar* spores collected at different time points after initiation of germination showed distinct transcriptional profiles (Sephton-Clark et al., 2018). Distinct gene clusters showed time-dependent expression in *R. delemar* in accordance with developmental stages of fungal growth. Most transcriptional changes occurred within the first hour of germination, followed by a period of “transcriptional consistency” during isotropic swelling. The transition to hyphal growth was marked by another shift in gene expression. Notably, hyphal growth of *R. delemar* was marked by an increase in transcripts involved in the reactive oxygen stress (ROS) response and respiration. Comparison to transcriptional data sets for *Aspergillus niger* showed similarities between the two fungi at the initiation of germination. However, there were transcriptional patterns for different metabolic processes that were uniquely regulated by *R. delemar* during isotropic and hyphal growth attributed by the authors to the duplicative nature of the *R. delemar* genome, which would require a more tightly regulated germination process (Sephton-Clark et al., 2018). This same study also uncovered the increased expression of lipid storage and localization genes during the dormant spore stage (Sephton-Clark et al., 2018). It is likely these genes play a role in providing energy to the fungus in nutritionally deprived environment.

Other omics-based studies have demonstrated a role for lipid metabolism in *Mucorales*. In a transcriptome study comparing the transcriptome from *R. oryzae* in a mycelial morphology to a pellet-like morphology, Xu Q. et al. (2018) found that genes involved in fatty acid metabolism were amongst the differentially regulated genes. The genomes of a high-lipid and low-lipid producing *M. circinelloides* strain were sequenced and compared to elucidate potential determinants of lipid production (Tang et al., 2015). Transcriptomic analysis of *Cunninghamella echinulata* has provided some insight into mechanisms of lipid metabolism, including its ability to utilize trehalose as carbon source to produce gamma-linolenic acid (GLA) and changes in lipid metabolism according to long-term high temperature changes (Li et al., 2018, 2019).

Cases of primary cutaneous infection caused by *Myrmicaria irregularis* have increased, predominantly in China (Lu et al., 2013; Rammaert et al., 2014). Unlike most mucormycoses, *M. irregularis* infection has been characterized by chronic disease that is limited to the dermal and subcutaneous tissues. However, there is little understanding as to how *M. irregularis* adapts to the hypoxic environment of the skin. A comparison of the transcriptome of *M. irregularis* in normoxic and hypoxic atmospheres identified possible genetic determinants that allow *M. irregularis* to adapt to hypoxic conditions. Genes involved in lipid metabolism and endocytosis activation were upregulated in response to hypoxic conditions. This result was of significant interest since *M. irregularis* infections are often found in facial skin lesions where sebaceous glands are abundant (Sebum of sebaceous glands have high fatty acid concentrations). Furthermore, genes involved in carbon metabolism were downregulated, leading the authors to hypothesize that *M. irregularis* uses intracellular lipid pools rather than carbohydrates as an energy source (Xu W. et al., 2018).

The fungal cell wall plays an important role in the ability of *Mucorales* to survive in harsh conditions and its detection by the host immune system. One of the many gene families that were expanded by the WGD in *R. delemar* are genes involved in cell wall biosynthesis. The *R. delemar* genome contains nearly double the number of chitin synthases (CHS) and chitin deacetylases (CDA) encoding genes of other dikaryotic fungi (Ma et al., 2009). The expansions of the CHS- and CDA-encoding genes were also demonstrated in the genomes of 29 additional *Mucorales* species across 10 genera (Chibucos et al., 2016; Corrochano et al., 2016; Prakash et al., 2017). Genomic and transcriptomic studies have elucidated different ways in which *Mucorales* maintains and changes cell wall integrity. For example, in response to oxidative stress, *R. oryzae* upregulates genes related to chitin catabolism, most likely to reduce the chitin composition in its cell wall and reduce ROS-mediated damage (Xu Q. et al., 2018). Compared to other stages of germination, the transcriptome of *R. delemar* dormant spores show high levels of transcripts involved in chitin processes, suggesting that the turnover or degradation of the fungal cell wall may be an important process in the survival and maintenance of dormant *R. delemar* spores (Sephton-Clark et al., 2018). As expected, genes involved in cell wall biosynthesis and composition are differentially expressed as *R. delemar* undergoes germination. The identification of these genes by transcriptome analysis could allow researchers to target germination of *R. delemar* and prevent invasive mucormycosis. The genomes of *Apophysomyces* species show a unique profile of carbohydrate active enzymes (CAZymes) responsible for the generation of *Apophysomyces* cell wall polysaccharides, which may represent a novel antifungal target (Prakash et al., 2017). Difference patterns in CAZymes repertoire was also observed in the genomes of *Mucor* species that differed in lifestyle (Lebreton et al., 2020). Notably, *Mucor* species involved in cheese ripening had less CAZymes in their genome compared to more pathogenic and clinical isolates.

Steroids are a major component of fungal plasma membranes. The ergosterol biosynthesis pathway is conserved in *R. delemar* with multiple copies present for approximately half of the genes (Ma et al., 2009), However, there still remains a lot unknown about what mediates gene expression chances for steroid biosynthesis in *Mucorales*, the role of steroids in the cell wall composition, and differences within *Mucorales* species. For example, there is no significant difference in the expression of genes involves in steroid biosynthesis between *M. irregularis* strains in hypoxic versus nomoxic conditions (Xu W. et al., 2018).

### Virulence and Pathogenicity

A combination of transcriptomics and comparative genomics have identified several potential novel virulence factors that can be targeted for mucormycosis therapy. Whole genome comparisons of virulent and avirulent strains of *M. circinelloides* identified nearly 800 genes that were truncated, discontiguous, or absent in the avirulent strain, suggesting that they may be candidate virulence factors (López-Fernández et al., 2018). Additionally, there are significant transcriptomic differences between virulent and avirulent strains of *M. circinelloides* undergoing germination inside macrophages. Specifically, genes involved in nutrition assimilation and metabolism were more highly expressed in the virulent strain than in the avirulent strain, which allows it to survive and germinate within a phagosome (Pérez-Arques et al., 2019). These processes appear to under the control of Aft1 and Atf2 transcription factors (Pérez-Arques et al., 2019).

Comparative genomics has allowed insight into the role of a family of spore coat proteins in host cell invasion. CotH3 has been identified as an invasin in *Mucorales* and treatment with anti-CotH3 antibodies has been shown inhibit endothelial cell invasion and protect mice from mucormycosis (Gebremariam et al., 2014). Comparing the genomes of multiple *Mucorales* strains showed that strains from species more commonly isolated from mucormycosis patients (*Mucor, Rhizopus, Rhizomucor, Cokeromyces*, and *Lichtheimia*) have 6–7 copies of the CotH-like genes (Gravesen, 1978; Michael et al., 2006) where strains isolated less frequently from infections (*Apophysomyces, Cunninghamella, Saksenae, Syncephalastrum*, and *Umbelopsis*) have 1–2 copies. In contrast, *Entomophorales* isolates, which are a taxa of fungi closely related to Mucoromycotina but cause superficial infections, did not contain any CotH-like genes (Chibucos et al., 2016). One recent study claims that *Apophysomyces* spp. based on the examination of 3 genomes, have >15 copies of CotH-like genes (Prakash et al., 2017). The discordant estimation of CotH-like gene copy number in *Apophysomyces* spp. between the two studies (2 in Chibucos et al., 2016 versus >15 in Prakash et al., 2017) likely reflects the methods used to identify the gene family and highlights how the biological interpretation of genomic studies can be strongly biased by the analytical methods used. Additionally, correlations between CotH mRNA abundance and virulence can also be observed when comparing multiple species within a genus. When comparing the transcriptome of *Mucor* isolates that vary in lifestyles and clinical relevance, Lebreton et al. (2019) observed that the number of CotH transcripts were higher in pathogenic strains of *Mucor* compared to *Mucor* strains utilized in production.

Secreted proteases are another class of virulence factor that have been described for fungal pathogens, including *Mucorales*. Protease gene families are one of the many gene families that are expanded due to the WGD of *R. delemar*, and may account for its invasive nature (Ma et al., 2009). The genome of *L. corymbifera, Mucor* species and *Apophysomyces* species also contain a significant number of predicted secreted proteases (Schwartze et al., 2014; Prakash et al., 2017; Lebreton et al., 2020). A clinical isolate of *M*ucor *velutinosus*, which was previously not thought to be a major cause of mucormycosis, showed the presence of unique secreted aspartyl proteases which contribute to skin dissemination (Shelburne et al., 2015).

The production of secondary metabolites by fungi may account for virulence by acting as a secreted toxin. In 2013, a food-born illness outbreak occurred after a batch of Chobani yogurt was contaminated with a mold that the FDA identified as *M. circinelloides*. Whole genome analysis of the strain that caused the outbreak (named Mucho) identified multiple genes predicted to have a role in the production of secondary metabolites, indicating that production of toxins by Mucho may have been the cause of the food-borne outbreak (Lee et al., 2014). However, a comparative transcriptome analysis of different *Mucor* strains showed there was no difference in the number of transcripts involved in secondary metabolites between pathogenic and non-pathogenic strains of *Mucor*. However, it is worth noting that this study was done on *Mucor* strains grown on PDA medium and not under conditions that more closely resemble an infection (Lebreton et al., 2019).

### Iron and Acquisition

Iron is a common currency required for survival for nearly all organisms. Microbes must utilize different pathways to acquire iron for growth and virulence. Commonly, microbes produce siderophores which are secreted from the organism to scavenge and bring back iron. However, sequencing of the *R. delemar* genome revealed the lack of non-ribosomal peptide synthetases (NRPs), which produce the most common siderophores used by microbes. Alternatively, the *R. delemar* genome encodes Rhizoferrin, a siderophore that collects free iron instead of serum-bound iron, and two copies of a gene encoding heme oxygenase (Ma et al., 2009). Transcriptomic analysis of *R. delemar* infected bone marrow derived macrophages (BMDM) showed an upregulation in iron acquisition genes, particularly *fet3*, a multicopper ferroxidase required for ferrous iron uptake and fungal dimorphism, and *ftr1*, a high affinity iron permease (Ibrahim et al., 2010; Andrianaki et al., 2018; Navarro-Mendoza et al., 2018). *Rhizopus* mutants with reduced *ftr1* copies showed reduced germination in BMDM phagosome following iron supplementation, demonstrating the essential role of iron acquisition for *Rhizopus* during macrophage infection (Andrianaki et al., 2018). Dormant *R. delemar* spores show an upregulation of genes involved in latter stages of iron-sulfur cluster biosynthesis compared to *R. delemar* undergoing germination. Initial phases of *R. delemar* germination are characterized by a rapid increase of iron acquisition transcripts (Sephton-Clark et al., 2018).

The genome of *L. corymbifera* contains multiple genes involved in iron acquisition, including multiple copies of the *ftr1*, which vary in levels of expression under iron limiting conditions (Schwartze et al., 2014). Transcriptomic analysis of *L. corymbifera* in iron-limiting conditions identified novel virulence factors, including potential transcription factors that act as key regulators in iron acquisition (Schwartze et al., 2014).

Similar to other *Mucorales* genomes, the *Apophysomyces* spp. lack genes encoding NRPs in their genomes and contain multiple genes involved in iron acquisition pathways, including genes in the reductive pathway and siderophores (Prakash et al., 2017). Sequencing of five different *Mucor* genomes from strains that represented different environments and lifestyles identified homologs of genes involved in different mechanisms of iron acquisition. Similar to *R. delemar*, the results suggested that *Mucor* species relied predominantly on Rhizoferrin for iron acquisition (Lebreton et al., 2020). However, the two strains associated with cheese production showed a reduced number of genes related to iron acquisition.

### RNAi Silencing Pathways in *Mucorales*

RNA interference pathways are highly conserved among eukaryotes as a way of negatively regulating gene expression through small non-coding RNAs or short RNAs (sRNAs) (Billmyre et al., 2013). The canonical RNAi pathway generates double stranded RNA (dsRNA) by RNA-dependent RNA polymerases (RdRPs), which are then processed by Dicer enzymes to produce the sRNAs. In turn, these endogenous sRNAs are used to repress various target sequences (Meister and Tuschl, 2004). High throughput sequencing of small RNAs from a wild-type *M. circinelloides* strain, a strain carrying a deletion in *RdRP1*, and a strain carrying a deletion in *DCL2*, which encodes a *M. circinelloides* Dicer gene, revealed the identify of a new class of endogenous small RNAs that map to exons and regulate the expression of protein-coding genes from where they were produced (named exonic siRNAs) (Nicolas et al., 2010). The impact of these exonic siRNAs was further characterized in a follow-up study that examined the mRNA transcriptome of these mutants during different stages of vegetative growth (Nicolás et al., 2015). Deletion of genes involves in the canonical RNAi silencing machinery resulted in significant mRNA accumulation during exponential and stationary growth phases of *M. circinelloides.* However, expression of many genes involved in processes such as growth at an acidic pH and sexual interaction were found to be unaltered in the RNAi machinery mutants, suggesting that these processes are regulated by a Dicer-independent non-canonical RNAi pathway (NCRIP) (Nicolás et al., 2015). Indeed, a NCRIP has recently been discovered in *M. circinelloides*, which relies on RdRP1 and R3B2, a novel RNase-III like protein required for cleavage activity. Transcriptomics on *rdrp1* and *r3b2* mutants demonstrated that these genes play important roles in regulating the fungal response to stressful environments, such as macrophage phagocytosis, as well as movement of TEs. These results that NCRIP play a role in controlling virulence in *M. circinelloides* (Pérez-Arques et al., 2020).

Recently, a novel role for RNAi machinery in *M. circinelloides* antifungal resistance has been elucidated through RNA sequencing on small RNAs. FK506 is an antifungal drug that interacts with the fungal FKBP12 isomerase, which inhibits protein phosphatase calcineurin, an important virulence factor in *M. circinelloides* that plays a key role in dimorphic transition. By blocking calcineurin, FK506 is able to block hyphal growth of *M. circinelloides* and restrict the fungus to yeast-phase growth (Calo et al., 2014). *M. circinelloides* can develop resistance to FK506 through Mendelian mutations in the *fkbA* gene, which encodes for FKB12. Additionally, *M. circinelloides* can exhibit a transient resistance to FK506 that is dependent on an epigenetic RNAi pathway. High throughput sequencing of small RNAs isolated from FK506-resistant epimutant strains of *M. circinelloides* revealed several sRNAs that are complementary to the *fkbA* mRNA (Calo et al., 2014). Establishment of these FK506-resistant epimutants is characterized by an abundance of these *fkbA*-targeting small RNAs to temporarily silence the expression of *fkbA* and prevent targeting by FK506 to inhibit hyphal growth.

This phenomenon was also observed in *M. circinelloides* epimutants that were resistant to 5-fluoroorotic acid (5-FOA), which is converted into a toxin by two genes, orotate phosphoribosyltransferase (*pyrF*) and orotidine-5′monophosphate decarboxylase (*pyrG*) in the uracil biosynthetic pathway (Chang et al., 2019). Sequencing of small RNAs from 5-FOA resistant mutants showed a significant increase in sense and antisense sRNAs against *pyrF* and *pyrG*, which correlated with reduced gene expression (Chang et al., 2019). The identification of transient 5-FOA resistant *M. circinelloides* epimutants, along with transient mutants against FK506, suggests that RNAi-dependent epimutation may be significant mechanism of antifungal resistance for *Mucorales*. More research is necessary to determine if this phenomenon is used by other species of *Mucorales* and how this mechanism might contribute the high rates of clinical antifungal resistance that are widely observed in *Mucorales* at large.

### Extracellular Vesicles

Extracellular vesicles (EV) are produced by nearly all cells and recent research has highlighted a role of fungal-derived EVs in cell-cell communication in fungi and pathogenesis (Joffe et al., 2016). These molecules can contain a variety of molecules, including small RNAs (Peres da Silva et al., 2015). Transcriptomic analysis on EVs from two clinical strains of *R. delemar* showed an abundance of extracellular small RNAs (exRNAs) that varied in types and length (Liu et al., 2018). Prediction programs for miRNA suggested that the majority of secreted miRNA targeted host genes, specifically those related to carbohydrate metabolism, secondary metabolite biosynthesis, and the two-component system. EVs containing exRNA have emerged as potential biomarkers in fungal infection (Peres da Silva et al., 2015, 2019). Some of the small EV-derived RNAs appear to be strain-specific, demonstrating their potential use in diagnostics (Liu et al., 2018).

## Insights Into the Host Response and Mechanisms

In addition to gaining a more complete understanding of *Mucorales* biology during infection, transcriptomics has provided insight into the host response during mucormycoses as well as important host-pathogen interactions that govern the progression of disease. RNA-seq analysis of airway epithelial cells infected with *R. delemar, R. Oryzae*, or *M. circinelloides* showed a significant enrichment of genes that are known to be targets of platelet derived growth factor receptor B (PDGFRB) signaling (Mucormycosis Statistics, 2018). This pathway was of significant interest due to the angioinvasive nature of mucormycosis and the its role in host cell invasion was confirmed with the use of small molecule inhibitors of PDGFRB in an *in vitro* infection.

Transcriptome analysis of murine lungs from early-stage infection (14 h) by *R. delemar* showed a significant enrichment of genes that are known to be targets of epidermal growth factor receptor (EGFR) signaling suggesting that the EGFR pathway was activated in response to *R. delemar.* Subsequent *in vitro* and *in vivo* infection experiments demonstrated that EGFR was indeed phosphorylated (activated) upon *Mucorales* infection and governed the ability of Mucorales to invade and damage host cells. Importantly, inhibition of EGFR by gefitinib, an FDA-approved small molecule inhibitor of EGFR phosphorylation, reduced the ability of *R. delemar* to invade host cells and increased survival in a murine model of pulmonary mucormycosis (Watkins et al., 2018).

Other laboratories have utilized transcriptomics in varying animal models to further characterize the host response during mucormycosis. Chamilos et al. (2008a) used a fruit fly model to show infection-induced gene regulation in flies infected with *R. oryzae* compared to uninfected. Many of these genes that were differentially regulated have homologs in humans. Notably, *R. oryzae* infected flies showed a down-regulation of genes involved in skeletal muscle repair and tissue reconstruction and an upregulation of immune-induced and stress response genes (Chamilos et al., 2008a). Additionally, López-Muñoz et al. (2018) utilized an adult zebrafish model to characterize the host response to *M. circinelloides*. In addition to confirming previously reported links between *M. circinelloides* sporangiospore size and virulence, the authors used transcriptomics to show a robust inflammatory response in response to *M. circinelloides*, which was characterized by upregulation of genes involved in pro-inflammatory cytokines, such as IL-1β, TNF-α, and IL-22, complement factors, peptidoglycan recognition proteins (PGRP) and iron homeostasis (López-Muñoz et al., 2018). This study also demonstrated that host genes related to lipid transport activity were significantly down-regulated during a *M. circinelloides* infection (López-Muñoz et al., 2018).

Host transcriptome analysis of *R. delemar* infected primary murine BMDMs confirmed the importance of iron acquisition for *Rhizopus*, with the expression several iron metabolism related genes differentially expressed over the course of the *Rhizopus* infection (Andrianaki et al., 2018). This expression pattern was consistent with an M2 activation program, which is in line with previous research demonstrating a role of iron metabolism in macrophage polarization (Ganz, 2012; Agoro et al., 2018). Additionally, transcriptome analysis of different mucormycosis causing species showed minor but important differences in host gene expression, such as IL-1β, CD40LG, and the PKC complex (Chibucos et al., 2016).

Another study examined the transcriptome response of a murine macrophage cell line (J774.1) following phagocytosis of *Mucorales* strains. Pérez-Arques et al. (2019) compared the macrophage responses to a virulent and an avirulent isolate of *M. circinelloides*. The virulent strain elicited specific proinflammatory and apoptotic responses while the avirulent strain did not induce a robust transcriptional response (Pérez-Arques et al., 2019). These results provide a compelling case for inflammation and macrophage apoptosis being involved in the progression of disease cause by *M. circinelloides*.

Host functional screens can also reveal important fungus-host interactions. Wang et al. (2018) screened a panel of 528 lymphoblastoid cell lines, each derived from a different individual human, for *M. circinelloides*-induced production of basic fibroblast growth factor 2 (FGF2). FGF2 is a growth factor with important roles in angiogenesis, cell survival, tissue repair, and other biological processes (Wang et al., 2018). A genome-wide association study (GWAS) identified single nucleotide polymorphisms (SNPs) that are highly associated with the ability of host cells to produce FGF2 in response to *in vitro* infection with *M. circinelloides*. The genes containing these SNPs represent candidate host factors that potentially govern the interaction between *Mucorales* and humans.

## Genome-Guided Diagnostic Development

A major unmet clinical need in the approach to mucormycoses is the lack of a test which allows early and accurate diagnosis. There are no biomarkers identified to detect mucormycoses (Kontoyiannis and Lewis, 2011). The established fungal diagnostics assays which target ß-D-glucan and galactomannan do not detect components of the *Mucorales* cell wall (Kontoyiannis and Lewis, 2011). The pathophysiology, mode of acquisition, and underlying risk factors for mucormycoses and aspergillosis are similar, yet the therapeutic approach to treating each of these diseases is very different. Complicating matters are the observations that (a) voriconazole administration (a frontline therapy to treat aspergillosis) is considered a risk factor for developing mucormycosis and (b) exposure to voriconazole can increase the virulence of *Mucorales* species (Lamaris et al., 2009; Kontoyiannis and Lewis, 2011; Millon et al., 2015). Rapid management of disease is further hindered because there are no specific symptoms allowing differentiation between mucormycoses and infections caused by other filamentous fungi (Tacke et al., 2014). Molecular detection tools are also few and have not undergone extensive clinical validation (Kontoyiannis and Lewis, 2011). As a result, routine clinical non-culture-based molecular-based methods as a single approach for mucormycosis diagnosis is not recommended (Kontoyiannis and Lewis, 2011). Early and accurate diagnosis remains the single most important barrier for improving survival of mucormycosis patients because early identification and treatment are critical before angioinvasion and necrosis become too extensive and dissemination occurs (Chamilos et al., 2008b; Kontoyiannis and Lewis, 2011). The benefits of early diagnosis include less extensive and disfiguring surgery for the removal of necrotized tissue since antifungal therapy alone, in the absence of surgical removal of the infected focus, is rarely curative (Kontoyiannis and Lewis, 2011; Millon et al., 2015).

Biopsy and culture from sterile sites are critical to distinguish mucormycoses from more common and more antifungal-sensitive molds, such as *Aspergillus* (Spellberg et al., 2005; Kontoyiannis and Lewis, 2011). Current diagnosis of mucormycosis relies heavily on morphological identification from cultures, radiology, and histopathology (Baldin et al., 2018). Immunohistochemical reagents that detect *Mucorales* in tissue are currently available, but these methods are limited in that they do not give species-level identification (Bernal-Martinez et al., 2013). Even when hyphae are seen by histopathology, fungal cultures are only positive in 50% of cases because of the fragile nature of aseptate hyphae, which are often damaged during tissue manipulation (Kontoyiannis and Lewis, 2011). These hyphal elements often accompany tissue necrosis and fungal angioinvasion (Spellberg et al., 2005; Kontoyiannis and Lewis, 2011).

The importance of early differentiation of *Mucorales* from other mold infections has generated great interest and need in the development of non-culture- and non-histopathology-dependent diagnostic tests such as detection of specific fungal antigens or nucleic acids by PCR. Whole genome sequencing of several isolates of several mucormycosis-causing species has uncovered a pan-*Mucorales* gene family that is not present in *Aspergillus* species (Gebremariam et al., 2014; Chibucos et al., 2016; Baldin et al., 2018). To this end, Baldin et al. (2018) have demonstrated that PCR amplification of CotH genes that are universally and uniquely present in *Mucorales* represent a promising target for a sensitive, reliable, and simple method for the early detection of mucormycosis (Gebremariam et al., 2014; Chibucos et al., 2016). Using just a single primer set, the CotH genes can be PCR-amplified from plasma, urine, and BAL samples 24 h post-infection from mice infected intratracheally with *R. delemar*, *R. oryzae*, *M. circinelloides*, *L. corymbifera*, and *Cunninghamella bertholletiae* as well as in urine samples of patients with proven mucormycosis (Baldin et al., 2018). Species-level identification is important because clinically relevant Mucorales (*Mucor*, *Rhizopus*, and *Lictheimia* species) show varying resistance to antifungals (Chalupova et al., 2014). Further comparative genomic studies are required to identify species- and genus-specific targets.

## Future Directions

The omics studies outlined here have provided valuable insights into many aspects surrounding mucormycosis and have collectively served as an exciting proof-of-principle that these types of approaches have the potential to uncover new and interesting biology, as well as clinically actionable information. Below, we discuss some of the major unanswered questions in the field as well as some exciting aspects of *Mucorales* biology that can be addressed using unbiased, systematic, genome-wide approaches. In each of the cases below, the omics approach has the potential to serve as a significant first step and should always be combined, if technically feasible, with more focused, functional follow-up studies.

Which fungal genes are expressed during infection? This fundamental question has been very difficult to address for technical reasons. Specifically, when extracting total RNA from a host sample that has been infected with any microbe, the signal from host transcripts typically overwhelms the signal from the infecting microbe and the pathogen RNA consists of only a tiny portion (0.1% or less) of the total RNA extracted. Enrichment strategies to selectively enrich fungal transcripts from a total RNA samples harvested from infected mouse tissues have been successfully applied to murine infection models of *C. albicans* (Amorim-Vaz et al., 2015) and *A. fumigatus* (Chung et al., 2018). Application of selective enrichment techniques to study *Mucorales* gene expression of isolates from different genera in the murine models of mucormycosis is sure to unearth important virulence genes that can ultimately serve as therapeutic targets or biomarkers for diagnostics.

Does the microbiome play a role in the establishment or progression of mucormycosis? To date only two studies have performed a microbiome survey in the context of infection (Shelburne et al., 2015; Mueller et al., 2019). Mueller et al. (2019) examined the gut microbiome (both bacterial and fungal) of mice infected with *M. circinelloides* in a model of gastrointestinal (GI) mucormycosis and found a significant decrease in the abundance of the bacteria *Akkermansia muciniphila*, a microbe known to be positively correlated with good health, in the GI tract following introduction of *M. circinelloides*. Given the recent connection between the gastrointestinal microbiome and antifungal immunity in the lung (Shao et al., 2019), it is conceivable that the gut microbiome may be relevant to progression of pulmonary mucormycosis as well as gastrointestinal mucormycosis. Shelburne et al. (2015) analyzed oral and fecal microbiomes of a single leukemia patient (AML) over the course of several weeks as they progressed through chemotherapy, development of neutropenia and to subsequent invasive mucormycosis infection caused by *M. velutinosus* which occurred amid a dysbiotic microbiome with low α-diversity, dominated by staphylococci. Many more studies are required to truly establish what role, if any, the microbiome plays in the interaction between *Mucorales* and the infected host.

How do bacterial endosymbionts of Mucorales affect disease establishment and progression? Both clinical and plant pathogenic isolates of Mucorales are known to harbor bacterial endosymbionts (Ibrahim et al., 2008). Some initial experiments suggested that the bacterial endosymbionts had no effect on the virulence potential of the fungus that they live inside of Ibrahim et al. (2008) but recent work has set a precedent for a role in evasion of host innate immune cells (Itabanga et al., 2019). Specifically, *Ralstonia pickettii* promotes the ability of *R. microsporus* to survive killing by macrophages (Itabanga et al., 2019). In a transcriptome-focused companion paper, Sephton-Clark et al. (2019) examined the transcriptional response of J774.1 macrophages following phagocytosis of *R. delemar* and *R. microsporus* either with or without their bacterial endosymbionts living inside them. This study revealed that endosymbiont-cured *R. microsporus* elicited a much stronger pro-inflammatory response than did *R. microsporus* which contained the bacterial endosymbiont, consistent with increased ability of macrophages to kill *R. pickettii*-cured *R. microsporus*. These two studies provide a clear and exciting mandate for a more wide-spread analysis of the *Mucorales*-endosymbiont associations to determine how generalizable this phenomenon is to other *Mucorales*-host interactions.

Mycoviruses (fungal viruses) and their effect on fungal hosts have been well-characterized in multiple fungal species, including fungi that are pathogenic to humans (Kotta-Loizou and Coutts, 2017). Of clinical interest are mycoviruses that increase or decrease fungal virulence (hypervirulence or hypovirulent, respectively) and confer a killer phenotype. Double-stranded RNA viral elements have been found in *Mucorales* species (Vágvölgyi et al., 1993, 1998; Papp et al., 2001); however, there have been little to no published attempts to further characterize these mycoviruses or identify novel *Mucorales* infecting mycoviruses. NGS-based approaches have already been utilized to discover and characterize mycoviruses in pathogenic fungi, including *Aspergillus* (Vainio et al., 2015; Zoll et al., 2018). This approach has already shown potential in the *Mucorales* field. Whole genome sequencing and phylogenomic comparison of *Rhizopus* species showed the presence of *pol* fragments from *Caulimovirus* (plant virus) in one-third of the analyzed genomes (Gryganskyi et al., 2018). Recently, a transcriptomic analysis of *M. irregularis* demonstrated the presence of a gene for a predicted RNA polymerase domain specific to a negative strand RNA virus (Barata et al., 2019). RNA sequencing has also led to the discovery of two *Narnavirus* members in *R. microsporus* in a novel fungal-bacterial-viral holobiont system (Espino-Vazquez et al., 2020). The role of these viruses in *Rhizopus* biology are still questioned but it is clear that they play a role of in *Rhizopus* biology. Infection of the viruses alone decreased asexual reproduction by reducing the number of *R. microsporus* sporangiospores. However, co-infection of these *Narnaviruses* with a *Mycetohabitans* bacterial symbiont speared to be required for successful sexual reproduction of *M. microsporus*.

As we continue to use -omics based approaches to characterize mucormycosis causing fungi, an emphasis should be placed on their respective mycoviruses to obtain a more complete understanding of their biology. Beyond providing novel information on potential symbioses, mycovirus therapy presents a novel therapeutic solution for IFI, especially as resistance to anti-fungal treatments are on the rise (Van De Sande et al., 2010). Success in the use of bacteriophage to treat respiratory bacterial infections suggest that parallel results could occur through the use of mycoviruses, which has been explored for *Aspergillus* therapy (van de Sande and Vonk, 2019; Takahashi-Nakaguchi et al., 2020). Further discovery and characterizations of novel mycoviruses in *Mucorales* through -omics based approaches will not only offer a more complete biology of these fungi but may identify potential use of mycovirus therapy for mucormycoses.

## Author Contributions

AS and VB developed the ideas, wrote, and edited the manuscript. TW wrote the manuscript. All authors contributed to the article and approved the submitted version.

## Conflict of Interest

The authors declare that the research was conducted in the absence of any commercial or financial relationships that could be construed as a potential conflict of interest.

## Footnotes

1 https://www.ncbi.nlm.nih.gov/genome
